# A Comparison of Porphyrin Photosensitizers in Photodynamic Inactivation of RNA and DNA Bacteriophages

**DOI:** 10.3390/v13030530

**Published:** 2021-03-23

**Authors:** Joe Heffron, Matthew Bork, Brooke K. Mayer, Troy Skwor

**Affiliations:** 1 Department of Civil, Construction and Environmental Engineering, Marquette University, 1637 W. Wisconsin Ave, Milwaukee, WI 53233, USA; joeheffron@gmail.com (J.H.); brooke.mayer@marquette.edu (B.K.M.); 2 Department of Chemical and Biological Sciences, Rockford University, 5050 E. State St., Rockford, IL 61108, USA; mbork@rockford.edu; 3 Department of Biomedical Sciences, University of Wisconsin–Milwaukee, 2400 E. Hartford Ave., Milwaukee, WI 53211, USA

**Keywords:** disinfection, blue light, metalloporphyrin, singlet oxygen, TMPyP, virus

## Abstract

Effective broad-spectrum antiviral treatments are in dire need as disinfectants and therapeutic alternatives. One such method of disinfection is photodynamic inactivation, which involves the production of reactive oxygen species from dissolved oxygen in response to light-stimulated photosensitizers. This study evaluated the efficacy of functionalized porphyrin compounds for photodynamic inactivation of bacteriophages as human virus surrogates. A blue-light light emitting diode (LED) lamp was used to activate porphyrin compounds in aqueous solution (phosphate buffer). The DNA bacteriophages ΦX174 and P22 were more resistant to porphyrin TMPyP photodynamic inactivation than RNA bacteriophage fr, with increasing rates of inactivation in the order: ΦX174 << P22 << fr. Bacteriophage ΦX174 was therefore considered a resistant virus suitable for the evaluation of three additional porphyrins. These porphyrins were synthesized from TMPyP by inclusion of a central palladium ion (PdT4) and/or the addition of a hydrophobic C14 chain (PdC14 or C14). While the inactivation rate of bacteriophage ΦX174 via TMPyP was similar to previous reports of resistant viruses, ΦX174 inactivation increased by a factor of approximately 2.5 using the metalloporphyrins PdT4 and PdC14. The order of porphyrin effectiveness was TMPyP < C14 < PdT4 < PdC14, indicating that both Pd^2+^ ligation and C14 functionalization aided virus inactivation.

## 1. Introduction

In the midst of a global pandemic, any case made for the grim impact of viruses on human health, let alone economies and political order, seems to be an understatement. However, beyond the devastation of the COVID-19 pandemic, viruses constitute a massive burden to public health. Viruses that can survive on surfaces and in water (primarily nonenveloped viruses) are responsible for outbreaks of both chronic and acute illnesses in recreational water (e.g., adenovirus, hepatitis A and E viruses [1]), drinking water (e.g., rotavirus [2] and enterovirus [3]), and food (e.g., norovirus [4]). Additionally, the release of bacteriophages from wastewater treatment facilities increases health risks due to their role in horizontal gene transfer of virulence genes [5], as well as heavy metal and antibiotic resistance genes [6].

Photodynamic inactivation of viruses has been suggested for diverse applications, including water/wastewater treatment [7,8,9], food preservation and aquaculture [10], antimicrobial polymers [11], and disinfection of blood and blood products [12,13,14]. Inactivation of viruses utilizing this mechanism is dependent on the presence of photosensitizers that are excited via photons, producing reactive oxygen species as a byproduct. One class of photosensitizers includes porphyrins, which are macrocyclic compounds that can act as ligands for metal ions—e.g., heme is a porphyrin that binds iron [15]. Similar in structure to the photosynthetic pigments chlorin and bacteriochlorin, porphyrins strongly absorb electromagnetic radiation in the visible spectrum. For this reason, porphyrins and their analogues are of interest for photochemical applications such as photodynamic inactivation (PDI) of pathogens [16].

Singlet oxygen-mediated oxidation is the primary mechanism of porphyrin-mediated PDI of viruses [17]. Singlet oxygen acts over short distances (~150 nm) in aqueous solutions and therefore reacts with targets close to the site of formation [11]. Due to porphyrin interactions with nucleic acid, proteins, and lipids [18], viral inactivation can be mediated through disrupting multiple viral targets—i.e., envelope lipids, capsid and core proteins [19], and nucleic acids [20,21].

Some studies [17,22,23,24] have demonstrated remarkably rapid virus inactivation by porphyrin PDI. However, these successes have been tempered by findings that the fastest rates are indicative only of some enveloped viruses and common bacteriophage surrogates [23,25,26,27,28]. Among nonenveloped viruses, inactivation of DNA viruses and even human single-stranded RNA (ssRNA) viruses is languid compared to ssRNA bacteriophages [23,25,26]. Therefore, PDI treatment processes must be evaluated using PDI-resistant viruses to ensure results are realistic for human viruses of concern. Further research is required to increase PDI efficacy for these resistant viruses.

Functionalized porphyrins may improve inactivation of PDI-resistant viruses by increasing singlet oxygen generation efficiency or enhancing sorption to the virion. In addition, using a narrow-spectrum, 405 nm light emitting diodes (LEDs) instead of white light to target the strongly absorbing Soret peak (~400 nm) of porphyrins may yield greater energy efficiency. The goal of this study was to evaluate PDI of bacteriophages employing functionalized porphyrins irradiated with a portable, 405 nm LED lamp. Three viruses (bacteriophages fr, P22, and ΦX174) were initially tested with a commonly used and promising porphyrin (TMPyP) [7,11,25]. Inactivation with different functionalized porphyrins (PdT4, C14, and PdC14) was further assessed against the most resistant bacteriophage (ΦX174). Lastly, we performed an efficacy comparison of our data to PDI of viruses utilizing various light sources and porphyrins from an array of studies to contextualize system performance.

## 2. Methods

### 2.1. Porphyrins and Metalloporphyrin Synthesis

Porphyrins 5,10,15,20-tetrakis (N-methylpyridinium-4-yl)porphyrin tetra(4-toluenesulfonate) (TMPyP) and meso-tri(N-methylpyridyl),meso-mono(N-tetradecylpyridyl)porphine tetrasulphonate (C14) were obtained from Frontier Scientific (Logan, UT, USA). Chelating palladium into the TMPyP or C14 porphyrin was performed following previous procedures [29,30]. Briefly, metalation occurred upon refluxing the sulfonate or tosylate salt of the water-soluble porphyrin in an aqueous solution containing ten-fold excess Pd^2+^ via a palladium intermediate, Pd(DMSO)_2_Cl_2_, for 2 days. Reaction progress was monitored using a GENSYS™10S UV–Vis Spectrophotometer (Thermo Scientific, MA, USA), and upon completion precipitation was induced by addition of KPF_6_ in acetonitrile. Ion exchange with tetrabutylammonium nitrate yielded the water-soluble nitrate product, which was isolated and further characterized in solution by molecular absorption spectroscopy (GENSYS™10S UV–Vis Spectrophotometer).

To determine the effect of inserting palladium into the porphyrin base (TMPyP), singlet oxygen production was measured following a previous method [29]. TMPyP and PdT4 were each irradiated at their corresponding Soret peak and singlet oxygen emission was quantified using a Fluorolog 3 Spectrofluorometer (Horiba, NJ, USA) between 1200 and 1280 nm (Supplementary Materials Figure S1) [31].

### 2.2. Bacteriophage Propagation

Three bacteriophages were used as model viruses: fr, P22, and ΦX174. The properties of these bacteriophages are summarized in Table 1. Bacteriophages were propagated using the double-agar layer (DAL) method [32], followed by two cycles of polyethylene glycol (PEG) precipitation, followed by a Vertrel XF (DuPont, Wilmington, DE) purification, as previously described by Mayer et al. [33].

### 2.3. Absorbance Spectra

Absorbance spectra were measured to determine the extent of porphyrin binding to bacteriophages. Binding of porphyrin to target macromolecules increases inactivation efficiency, though binding may not be necessary for inactivation [8,14,39]. Porphyrin was added to Buffered Demand Free (BDF, pH 7.0) solution containing no bacteriophage (control) or a high titer of bacteriophage fr, P22, or ΦX174, independently. Porphyrin and bacteriophages were incubated in the dark at room temperature for durations of 5, 15 or 60 min. Absorbance spectra were then measured using a Lambda 35 UV–Vis spectrophotometer (PerkinElmer, Waltham, MA) at a scan rate of 960 nm/min and resolution of 1 nm. Porphyrin concentrations used for PDI in this study (10 µM) were assumed to be in excess of the binding capacity due to no shift in PdT4 Soret peak after 60-min incubation, even with a titer of bacteriophages (~10^8^ PFU/mL) far higher than that used for PDI experiments (Figure S4).

### 2.4. Photodynamic Inactivation

Bacteriophages fr, P22, and ΦX174 were spiked into 0.01 M BDF at approximately 10^6^ PFU/mL Concentrated porphyrin was added to 2 mL of bacteriophage solution for a final concentration of 10 µM. The PDI procedure was adapted from Skwor et al. [29]. Briefly, porphyrins were allowed to incubate with bacteriophages in the dark at room temperature for 5 min. The sample was then illuminated by a 405 nm blue-light LED (WARP, Quantum Devices, Inc., OH, USA) at 60 mW cm^-2^ for varying durations (1–352 s). The short duration of exposure imbued slight variations in timing due to manual operation of the handheld LED lamp (e.g., for 1 sec vs. 2 sec, it was inherently difficult to maintain accuracy). After treatment, samples were quenched with an excess of sodium thiosulfate (0.1 M) to halt any additional reactions, and serial diluted into BDF at pH 7.0. Samples (10 µL) of each dilution were plated onto 0.7% tryptic soy agar containing phage-specific hosts using the spot titer plaque assay method, as described by Beck et al. [40]. Plating and incubation were carried out in a darkened room with minimal ambient lighting. Controls were evaluated to determine inactivation of phages via porphyrin in ambient light (darkened room) and via blue light in the absence of porphyrin. No bacteriophages were inactivated by blue light alone (Figure S2). Only bacteriophage fr showed minor inactivation (<1 log_10_) from dark exposure to porphyrin (Figure S3). Log_10_ inactivation was calculated using the ambient light porphyrin control as a baseline to best represent inactivation due to the porphyrin/blue-light process.

### 2.5. Data Analysis

Pseudo-first order inactivation rates were determined by least squares regression of log_10_ reduction in virus concentrations over time. Type III ANOVA was used to determine the significance of interactions (i.e., differences in inactivation rates with different porphyrins) using the car::Anova function in R [41]. Post hoc Tukey pairwise comparisons were used to determine significant differences between individual conditions (e.g., two different porphyrins or pH conditions) using the lsmeans::lsmeans function in R [42].

## 3. Results and Discussion

### 3.1. Confirmation of Free and Bound Porphyrin in Test Conditions

**Figure 1:**
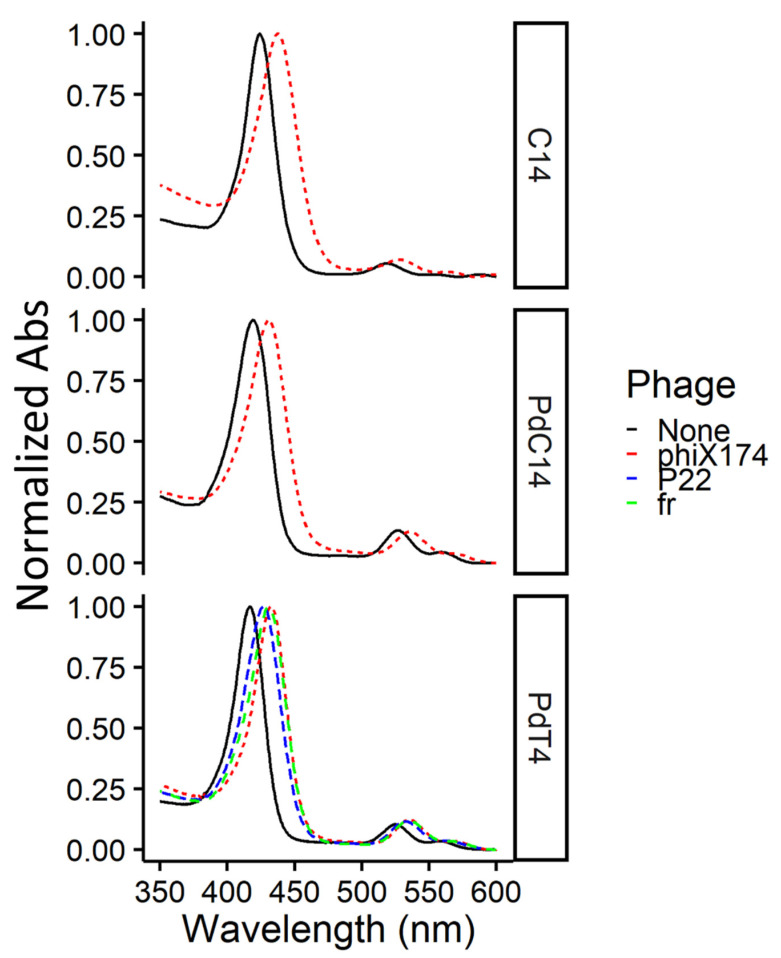
Normalized absorbance spectra for porphyrins C14, PdC14, and PdT4 (~2 µM) alone and with bacteriophages fr, P22, or ΦX174 (~10^10^ PFU/mL) after 5-min dark incubation at room temperature. Due to slightly varying porphyrin concentrations, spectra were normalized to maximum absorbance values within the displayed range (350–600 nm) to clearly depict the shift in the Soret peak. All porphyrin spectra show a shift in the Soret peak after incubation with bacteriophages (dashed lines), indicating porphyrin-phage binding.

To confirm the presence of both free and bound porphyrin at concentrations used in this study (i.e., an excess of porphyrin), spectra were measured for the metalloporphyrin PdT4 both with and without bacteriophage. At a low PdT4:virus ratio (~2 µM PdT4: ~10^10^ PFU/mL), a noticeable shift was observed in the PdT4 Soret peak after 5 min incubation (Figure 1). This red shift is caused by porphyrin binding, most likely to bacteriophage capsid proteins, though the shift is evident with nucleic acid and lipids as well [18]. No further shift was observed between 5- and 15-min incubation periods (Figure S5). Binding of bacteriophage ΦX174 to C14 and PdC14 was also confirmed within 5 min (Figure 1). Therefore, the 5-min incubation period used in this study was sufficient to achieve porphyrin binding.

### 3.2. Relative Resistance of Bacteriophages to PDI

**Figure 2:**
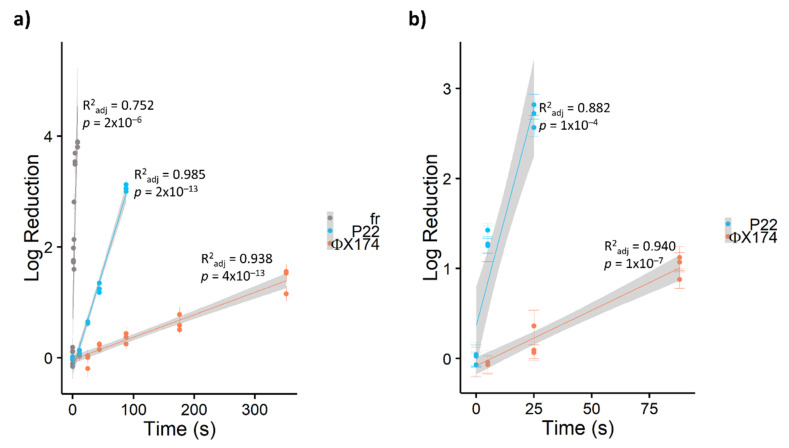
Log_10_ reduction in bacteriophages via a) TMPyP- and b) PdT4-mediated blue LED photoinactivation. Bacteriophage fr is shown in a) as an example of extremely rapid inactivation of RNA bacteriophages. All points represent single experimental replicates (mean of 10 plaque counts). Error bars represent ± 1 standard deviation of measurement. Shaded regions represent 95% confidence intervals for the least-squares linear regressions.

In this study, bacteriophages showed widely divergent susceptibility to PDI, with inactivation rates varying by orders of magnitude from ΦX174 << P22 << fr (Table 2). As shown in Figure 2, ssRNA bacteriophage fr was inactivated by approximately 4 log_10_ within 10 seconds (~0.5 J cm^−2^) by TMPyP, while ssDNA bacteriophage ΦX174 required over one minute (≥5 J cm^−2^) for a single log_10_ inactivation with any porphyrin. Previous studies [23,43] have reported a similar divergence in susceptibility to PDI among bacteriophages. Comparing PDI of seven bacteriophages in the presence of cationic porphyrin Tri-Py+-Me-PF, Costa et al. [23] found that DNA bacteriophages required 90–180 minutes to achieve 4-log inactivation, while 4-log inactivation of RNA bacteriophages occurred within 15 minutes. In a study of inactivation via UV-activated fullerenes, Hotze et al. [43] also found that bacteriophage MS2 (ssRNA) was inactivated more rapidly than the dsDNA phages PRD1 and T7. Thus, increased resistance was attributed to viruses with DNA genomes. However, Majiya et al. [25] determined that inactivation via TMPyP was not similarly effective in inactivating murine norovirus or bovine enterovirus, ssRNA viruses with animal hosts. In addition, not all DNA viruses are equally resistant. In our study, tailed bacteriophage P22 (dsDNA) was inactivated approximately ten times faster via PDI than ΦX174 (ssDNA). Similarly, a T4-like bacteriophage (dsDNA) in Costa et al.’s study [23] was inactivated approximately two- to four-fold faster than other dsDNA bacteriophages. Therefore, bacteriophage susceptibility to PDI is multifactorial, and the type of nucleic acid is not the sole determiner of virus resistance.

Known ssRNA bacteriophages fall into the classification of *Leviviridae*, and share some idiosyncratic traits. Many ssRNA phages are assembled from electrostatically bound RNA/capsid subunits and feature large regions of the capsid devoted to electrostatic binding of the genome polynucleotide [44,45]. Schneider et al. [46] determined that protein-RNA cross-linking was a significant mechanism of inactivation of *Leviviridae* phage Qβ via methylene blue PDI. By contrast, TMPyP PDI was not found to destabilize the DNA-protein interaction in dsDNA T7 phage, [39] whose genome is spooled into the procapsid (i.e., without extensive electrostatic interaction with the capsid) [47]. Therefore, large, closely associated RNA-capsid regions may be the reason for faster inactivation rates in ssRNA bacteriophages due to the closer proximity of reactive oxygen intermediates post-irradiance. *Leviviridae* also feature a single copy of a maturation protein, which is essential for viral attachment and penetration. Majiya et al. [17] determined that this maturation protein was the primary target of TMPyP on MS2 phages. Thus, the maturation protein may represent another common weakness of *Leviviridae* phages to PDI. As shown in Table 2, nonenveloped human RNA viruses are not inactivated as rapidly as RNA phages, but rather at rates similar to those of DNA phages (10^−4^–10^−7^ L cm^2^ µmol^−1^ mJ^−1^). Therefore, the extreme susceptibility of ssRNA phages to PDI is not due to RNA genome alone, and ssRNA phages make poor surrogates for PDI treatment of human viruses of concern.

Cationic porphyrin-mediated PDI is facilitated by binding of porphyrin to the virion, which may occur on either capsid proteins or polynucleotides [39]. Casteel et al. [14] showed that cationic TMPyP was more effective than an anionic porphyrin for inactivating bacteriophage MS2 (pI ~ 3.5) and hepatitis A virus (pI ~ 2.8) [38]. The isoelectric point of ΦX174 is 6.6 (indicative of net neutral virus charge at pH 6.6) [38], which is near the test conditions of this study (pH 7). Cationic porphyrins would be expected to have a greater attraction to the more negatively charged bacteriophages fr and P22, which have more acidic isoelectric points (Table 1) compared to ΦX174. Previous research [52,53] found that ΦX174 was similarly resistant to inactivation due to positively charged ferrous iron and polyaluminum chloride compared to bacteriophages with acidic isoelectric points. In addition, coulombic neutralization of basic capsid residues in genome-binding regions of *Leviviridae* capsids also results in an overall negative charge on the viral particle at circumneutral pH [44]. This negative charge may lead to greater attraction between the *Leviviridae* phages and cationic porphyrins. Many human RNA viruses also have isoelectric points near neutral [38,44], possibly contributing to the similar resistance of human RNA viruses and DNA viruses compared to RNA bacteriophages.

Since PDI tests were conducted at pH 7, near ΦX174’s isoelectric point, it is also possible that formation of virion aggregates contributed to virus resistance. Aggregation can cause viruses to be more resistant to inactivation because virions at the aggregate interior are protected from highly reactive oxidants [54]. To evaluate the potential impact of ΦX174 aggregation on PDI, filtered (0.2 µm) bacteriophage ΦX174 stock was added to BDF solutions at pH 5 or pH 8 and allowed to equilibrate overnight at 4 °C. The rate of ΦX174 inactivation was not significantly different between pH 7 and either pH 5 or pH 8 (*p* > 0.25, data not shown). Therefore, aggregation is unlikely to be responsible for ΦX174 resistance.

### 3.3. Comparative Efficacy of Different Porphyrins for Virus Inactivation

**Figure 3:**
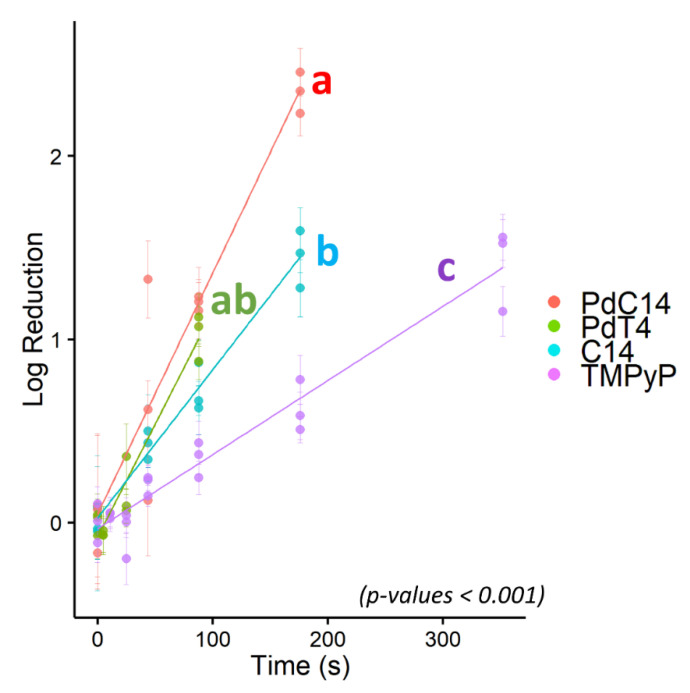
Log_10_ reduction of bacteriophage ΦX174 via blue-light photodynamic inactivation mediated by four porphyrins: PdC14, PdT4, C14, and TMPyP. All points represent single experimental replicates (mean of 10 plaque counts) and error bars represent ± 1 standard deviation of measurement. Porphyrins with statistically distinct inactivation rates are indicated by different letters, where PdT4 (“ab”) was not distinct from either PdC14 (“a”) or C14 (“b”). PdT4, C14, and PdC14 were distinct from TMPyP (“c”). *p* < 0.001.

Due to its resistance to PDI using porphyrin TMPyP, bacteriophage ΦX174 served as a model virus for testing additional modified porphyrins—C14 and PdC14. Previous studies have demonstrated enhanced oncolytic activity and bactericidal effects with the addition of Pd [29,55] and lipophilicity [56]. As shown in Figure 3, porphyrin effectiveness followed the order of TMPyP < C14 < PdT4 < PdC14. In a post hoc pairwise comparison of ΦX174 inactivation, PdT4 did not differ significantly from C14 (*p* = 0.11) or PdC14 (*p* = 0.19), but all other comparisons were significantly different (*p* < 0.001). PdT4 was similarly more effective than TMPyP for inactivating bacteriophage P22, as shown in Figure 2. These trends demonstrate that both inclusion of Pd in the metalloporphyrins and the functionalization of porphyrin with hydrophobic carbon chains appeared to increase porphyrin effectiveness. Palladium offers a kinetically stable square planar complex, and its size increases the rate of spin–orbit coupling, thus enhancing intersystem crossing to the triplet state (S1) [30]. Moreover, insertion of palladium into the porphyrin core induces a hypsochromic shift in the Soret band, which contributes to better overlap with the 405 nm LED source.

In addition, functionalizing the porphyrin with a hydrophobic C14 chain may have led to increased virion contact. Increasing cation hydrophobicity would help improve porphyrin–virion contact when electrostatic interactions were not favorable, as for bacteriophage ΦX174 at circumneutral pH. Previous research [57] found that increasing the hydrophobicity of photosensitizing compounds can increase their affinity for proteins over DNA, making them more selective for degradation of the viral capsid rather than the genome. Though porphyrins show evidence of DNA intercalation [30,39], the main targets of PDI tend to be on microbial surfaces—i.e., virus capsid proteins (only nonenveloped viruses are considered here) [19]. In addition, singlet oxygen reacts far more rapidly with protein residues than with nucleic acid (by ~1 to 3 orders of magnitude) [22,58,59]. Oxidation via singlet oxygen induces cross-linking of capsid proteins to one another, to the viral genome, and even between capsids [17,43,46]. Accordingly, Zupán et al. [12] found that inactivation of bacteriophage T7 was more effective with free porphyrin than DNA-intercalated porphyrin. Therefore, C14 functionalization may better target the vulnerable region of the virion (the capsid) and/or better target the virion itself. 

Addition of the nonpolar C14 tail to the porphyrin may also encourage the formation of aggregates. Pasternack et al. [60] concluded that TMPyP does not self-aggregate in water, which was attributed to the electrostatic interactions of a tetra-cationic species. Nonaggregation of the tetracationic species was further confirmed via nuclear magnetic resonance by Kano et al. [61], although the tri- and di- species did form dimers. Accordingly, it is possible that C14 self-aggregated due to the reduction in charge and amphiphilic nature of the chromophore (notably, Kano et al. used 500 times higher porphyrin concentrations than those used in the present study). The impact of this aggregation on PDI is unclear but may explain some of the variation in ΦX174 inactivation observed in Figure 3.

Another porphyrin-dependent mechanism associated with the inactivation of viruses is independent of light. Cationic porphyrins can interact with G-rich sequences capable of disrupting secondary structures such as G-quadruplexes [62,63]. These G-rich consensus sequences have been identified in numerous RNA and DNA animal viruses [64,65,66]. Rapozzi et al. [56] demonstrated a stronger PDI effect on G4-RNA degradation, as well as diminished translational activity due to disrupting secondary structure formation in a light-independent manner, with C14 compared to TMPyP. In the present study, minor inactivation was observed only for bacteriophage fr due to incubation with porphyrin without blue-light exposure (Figure S3). Therefore, light-independent inactivation was not a likely mechanism in this study.

### 3.4. Efficacy of Bacteriophage Inactivation Compared to Similar Techniques

The blue-light/porphyrin photodynamic virus inactivation demonstrated in this study compares favorably with other reports. In particular, previous studies have reported pseudo-first order rate constants in the range of 10^−3^–10^−4^ s^-1^ for DNA viruses; in this study, the most resistant DNA bacteriophage (ΦX174) had pseudo-first order rate constants from 10^−2^–10^−3^ s^−1^ (Table 2). In an attempt to control for the different conditions used in each experiment, the pseudo-first order rate constants were divided by the concentration (µM) and light intensity (mW cm^−2^) used in the study. This is not a perfect comparison, because in some studies (including this one), porphyrin may have been added in excess, thus resulting in a lower normalized inactivation rate. Nevertheless, after normalizing for light intensity and porphyrin concentration, the pseudo-first order ΦX174 inactivation rate observed here for TMPyP was similar to rates previously reported for (nonenveloped) DNA viruses and human RNA viruses (~10^−6^ to 10^−7^ L cm^2^ µmol^−1^ mJ^−1^). However, normalized ΦX174 inactivation rates for metalloporphyrins PdT4 and PdC14 were about an order of magnitude faster than previous reports (~10^−5^ L cm^2^ µmol^−1^ mJ^−1^), indicating significant improvement over TMPyP-mediated inactivation. Schagen et al. [67] reported nearly as rapid inactivation of recombinant adenovirus via methylene blue/halogen light inactivation, with a low concentration (1.3 µM) of methylene blue resulting in a greater normalized inactivation rate (~10^−4^ L cm^2^ µmol^−1^ mJ^−1^). However, the quantification of recombinant adenovirus via green fluorescent protein expression [67], rather than infectious cultural assay, may undermine direct comparison.

Single-stranded RNA bacteriophage fr was also inactivated at a rate similar to that of the fastest previously reported MS2 inactivation (Table 2). However, the rate of inactivation of ssRNA bacteriophages was so rapid in this study as to be difficult to quantify. (MS2 was initially investigated in this study, but plaques were not observed at any exposure, indicating inactivation beyond the quantifiable limits, even at short treatment times; therefore, the closely related bacteriophage fr was used instead). As discussed in Section 3.2, ssRNA phages are not useful model viruses for PDI processes. Future research into PDI and other nascent disinfection technologies should avoid relying on ssRNA bacteriophages as the default surrogates to avoid overstating inactivation results.

## 4. Conclusions

Viral emergence in the beginning of the 21st century, including the viruses responsible for COVID-19, SARS, MERS, Ebola, and Zika [68], stresses the importance of finding broad-spectrum antiviral treatments. Our study evaluated the inactivation efficacy of PDI with three novel photosensitizers against bacteriophages. We compared inactivation rates using our PDI light source and photosensitizers to an extensive group of virus photoinactivation studies to contextualize system performance. The results of this study led to the following conclusions:

- Both inclusion of a hydrophobic C14 chain and/or a central palladium ion in the porphyrin structure significantly improves bacteriophage inactivation using low-dose 405 nm illumination.
- Inactivation rates differed by two orders of magnitude among bacteriophages. Susceptibility to PDI is not entirely predictable by genome type; however, ssRNA bacteriophages are particularly susceptible and are therefore poor surrogates for human viruses of interest. In this study, bacteriophage ΦX174 was the most resistant, with inactivation rates comparable to human viruses of interest in the literature. Therefore, ΦX174 should be considered as a virus surrogate for PDI research.
- Inactivation rates using the novel PdT4, PdC14, and C14 porphyrins and blue-light LED were favorable in comparison with previous reports in the literature.

Future research is needed to evaluate the effectiveness of diverse functionalized porphyrins with various chelated metals against microbial targets. Even if a universally applicable “silver bullet” photosensitizer cannot be found, a toolbox of functional groups may allow design of targeted porphyrins for specific applications. Targeted PDI would have applications both in therapeutics and disinfection of resistant organisms in water and on surfaces.

## Figures and Tables

**Table 1:** Properties of bacteriophages used in this study, adapted from Mayer et al. [34] or as cited.

Bacteriophage	Host Bacteria	Baltimore Classification	Diameter (nm)	Isoelectric Point
fr (ATCC 15767-B1)	*E. coli* (ATCC 19853)	IV ((+)ssRNA)	19–23	3.5 [35]
P22 (ATCC 19585-B1)	*Salmonella enterica* subsp. *typhimurium* LT2 (ATCC 19585)	I (dsDNA)	52–60 [36]	3.4 [37]
ΦX174 (ATCC 13706-B1)	*E. coli* (ATCC 13706)	II (ssDNA)	23–27	6.0–7.0 [38]

**Table 2:** Comparison of visible light photodynamic virus inactivation across this and other studies.

Source	Light	Intensity (mW cm^−2^)	Compound *	Conc. (µM)	Virus	Genome	Pseudo-First Order Rate Constant (log_10_ s^−1^)	Normalized Rate Constant (log_10_ L cm^2^ µmol^−1^ mJ^−1^)
Enveloped mammalian viruses								
Obrien 1992 [48]	White (fluorescent)	7	Merocyanine 540	26	Herpes simplex 1 virus	dsDNA	2 × 10^−2^	9 × 10^−5^
Käsermann 1997 [49]	White (mercury)	29	Fullerene	1400	Vesicular stomatitis virus	ssRNA	5 × 10^−4^	1 × 10^−8^
					Semliki Forest virus	ssRNA	5 × 10^−4^	1 × 10^−8^
Moor 1997 [24]	Red (halogen)	46	AlPcS4	1	Vesicular Stomatitis virus	ssRNA	1 × 10^−2^	3 × 10^−4^
			Pc4	0.005			7 × 10^−3^	3 × 10^−2^
Silva 2005 [50]	White	50	Pyridylvinyl-substituted tetraphenol porphryin	0.5	Herpes simplex 1 virus	dsDNA	2 × 10^−3^	9 × 10^−5^
Peddinti 2008 [11]	White (400-700 nm)	80	ZnTMPyP	1% wt film	Vesicular Stomatitis virus	ssRNA	2 × 10^−3^	NA
Nikolaeva-Glomb 2017 [51]	Red (laser, 635nm)	100	Hematoporphyrin	20	Influenza virus A	ssRNA	6 × 10^−4^	3 × 10^−7^
					Bovine viral diarrhea virus	ssRNA	2 × 10^−3^	8 × 10^−7^
			GaPc1				1 × 10^−3^	6 × 10^−7^
			GaPc2				2 × 10^−3^	1 × 10^−6^
			InPc1				2 × 10^−3^	8 × 10^−7^
Remichkova 2017 [26]	Red (laser, 635nm)	100	ZnPcMe	0.58	Bovine viral diarrhea virus	ssRNA	7 × 10^−3^	1 × 10^−4^
					Herpes simplex 1 virus	dsDNA	1 × 10^−2^	2 × 10^−4^
					Vaccinia virus	dsDNA	7 × 10^−3^	1 × 10^−4^
					Newcastle disease virus	ssRNA	0	0
			ZnPcS		Bovine viral diarrhea virus	ssRNA	2 × 10^−2^	3 × 10^−4^
					Herpes simplex 1 virus	dsDNA	1 × 10^−2^	2 × 10^−4^
					Vaccinia virus	dsDNA	7 × 10^−3^	1 × 10^−4^
					Newcastle disease virus	ssRNA	3 × 10^−3^	6 × 10^−5^
Nonenveloped mammalian viruses								
chagen 1999 [52]	White (halogen)	106	Methylene blue	1.3	Recombinant adenovirus (E1 deficient)	dsDNA	2 × 10^−2^	1 × 10^−4^
			Rose bengal	10			7 × 10^−3^	6 × 10^−6^
			Uroporphyrin	20			5 × 10^−3^	2 × 10^−6^
			AlPcS4	10			3 × 10^−3^	3 × 10^−6^
Peddinti 2008 [11]	White	80	ZnTMPyP4+	1% wt film	Human adenovirus 5	dsDNA	1 × 10^−3^	NA
Nikolaeva-Glomb 2017 [51]	Red (laser, 635nm)	100	GaPc1	20	Human adenovirus 5	dsDNA	2 × 10^−3^	8 × 10^−7^
					Poliovirus 1	ssRNA	1 × 10^−3^	6 × 10^−7^
			GaPc2		Human adenovirus 5	dsDNA	2 × 10^−3^	1 × 10^−6^
					Poliovirus 1	ssRNA	6 × 10^−4^	3 × 10^−7^
			Hematoporphyrin		Human adenovirus 5	dsDNA	1 × 10^−3^	6 × 10^−7^
					Poliovirus 1	ssRNA	6 × 10^−4^	3 × 10^−7^
			InPc1		Human adenovirus 5	dsDNA	1 × 10^−3^	6 × 10^−7^
					Poliovirus 1	ssRNA	6 × 10^−4^	3 × 10^−7^
Remichkova 2017 [26]	Red (laser, 635nm)	100	ZnPcMe	0.58	Coxsackievirus B1	ssRNA	0	0
					Human adenovirus 5	dsDNA	8 × 10^−4^	1 × 10^−5^
Majiya 2018 [25]	White (fluorescent)	32	TMPyP	5	Murine norovirus-1	ssRNA	2 × 10^−3^	1 × 10^−5^
				10	Bovine enterovirus-2	ssRNA ssRNA	2 × 10^−3^	5 × 10^−6^
				5			3 × 10^−4^	2 × 10^−6^
				10		ssRNA	8 × 10^−4^	3 × 10^−6^
Nonenveloped bacteriophages								
Cho 2010 [22]	White (fluorescent)	0.2	Amine-functionalized fullerol	20	MS2 phage	ssRNA	1 × 10^−3^	3 × 10^−4^
	Sunlight	0.19		10			1 × 10^−3^	6 × 10^−4^
Costa 2012 [23]	White (fluorescent)	40	Tri-Py+-Me-PF	5	T4-like phage	dsDNA	7 × 10^−4^	4 × 10^−6^
				5	Aeromonas phage	dsDNA	2 × 10^−4^	9 × 10^−7^
				5	Vibrio phage	dsDNA	4 × 10^−4^	2 × 10^−6^
				5	Pseudomonas phage	dsDNA	4 × 10^−4^	2 × 10^−6^
				0.5	MS2 phage	ssRNA	4 × 10^−3^	2 × 10^−4^
				0.5	Qbeta phage	ssRNA	4 × 10^−3^	2 × 10^−4^
				0.5	LAIST_PG002 phage	ssRNA	7 × 10^−3^	3 × 10^−4^
Majiya 2018 [25]	White (fluorescent)	32	TMPyP	0.5	Qbeta phage	ssRNA	2 × 10^−2^	1 × 10^−3^
Majiya 2019 [7]	White (fluorescent)	32	TMPyP	0.5	MS2 phage	ssRNA	2 × 10^−1^	1 × 10^−2^
This study	Blue (LED, 405 nm)	60	TMPyP	10	ΦX174 phage	ssDNA	4 × 10^−3^	7 × 10^−6^
					P22 phage	dsDNA	4 × 10^−2^	6 × 10^−5^
					fr phage	ssRNA	5 × 10^−1^	8 × 10^−4^
			PdT4		ΦX174 phage	ssDNA	1 × 10^−2^	2 × 10^−5^
					P22 phage	dsDNA	1 × 10^−1^	2 × 10^−4^
			C14		ΦX174 phage	ssDNA ssDNA	8 × 10^−3^	1 × 10^−5^
			PdC14				1 × 10^−2^	2 × 10^−5^

* Guide to compound abbreviations (adopted from original authors): phthalocyanine-based: Pc4: phthalocyanine; PcS4: phthalocyanine tetrasulfonate; PcMe: 2,9,16,23-tetrakis(3-methylpyrydyloxy) phtalocyanine; PcS: 2,9,16,23-tetrakis(4-sulfophenoxy)phthalocyanine; GaPc1: tetra-methylpyridyloxy-substituted Ga phthalocyanine; GaPc2: octa-methylpyridyloxy-substituted Ga phthalocyanine; InPc1: tetra-methylpyridyloxy-substituted In phthalocyanine. Porphyrins: C14: meso-tri(N-methylpyridyl),meso-mono(N-tetradecylpyridyl)porphine tetrasulphonate; TMPyP: 5,10,15,20-tetrakis(N-methylpyridinium-4-yl)porphyrin tetra(4-toluenesulfonate); Tri-Py+-Me-PF: 5,10,15-tris(1-methyl- pyridinium-4-yl)-20-(pentafluorophenyl)porphyrin tri-iodide. Data for single-stranded RNA bacteriophages, which tend to be more susceptible to photodynamic inactivation than other viruses, are emphasized in red.

## Data Availability

The data presented in this study are openly available in the Supporting Materials and via FigShare at https://figshare.com/projects/Heffron_Bork_Mayer_Skwor_-_A_Comparison_of_Porphyrin_Photosensitizers_in_Photodynamic_Inactivation_of_RNA_and_DNA_Bacteriophages/100859, accessed on 5 February 2021.

## Supplementary Materials

The following are available online at https://www.mdpi.com/1999-4915/13/3/530/s1: Figure S1. Singlet oxygen emission post-illumination of porphyrins. Figure S2. Porphyrin and blue-light control tests. Figure S3. Porphyrin/phage binding indicated by absorbance spectra of PdT4 and bacteriophages. Figure S4. Calculation of inactivation rate constants from the literature.
