# Supplementary material for: A Comparison of Porphyrin Photosensitizers in Photodynamic Inactivation of RNA and DNA Bacteriophages
Source: Viruses. 2021 Mar 23;13(3):530. doi: 10.3390/v13030530 (PMC8005208; doi:10.3390/v13030530)
Supplement: Supplementary file 1 [file viruses-13-00530-s001.pdf]

Figure S1. Singlet oxygen emission post illumination of porphyrins. Equal concentrations of porphyrins were irradiated with the same intensity of light but at their corresponding Soret peak. Steady state oxygen emission was determined between 1220 and 1330 nm, and absorbance was corrected. As shown in Figure S1, PdT4 showed enhanced singlet oxygen generation over the same porphyrin without a metal ion (TMPyP) at their relative Soret peaks. The addition of the palladium ion enhances spin-orbit coupling,[1] resulting in greater singlet oxygen generation. Since photophysical properties are mostly dictated by the porphyrin ring, we would expect a similar relationship between C14 and PdC14.

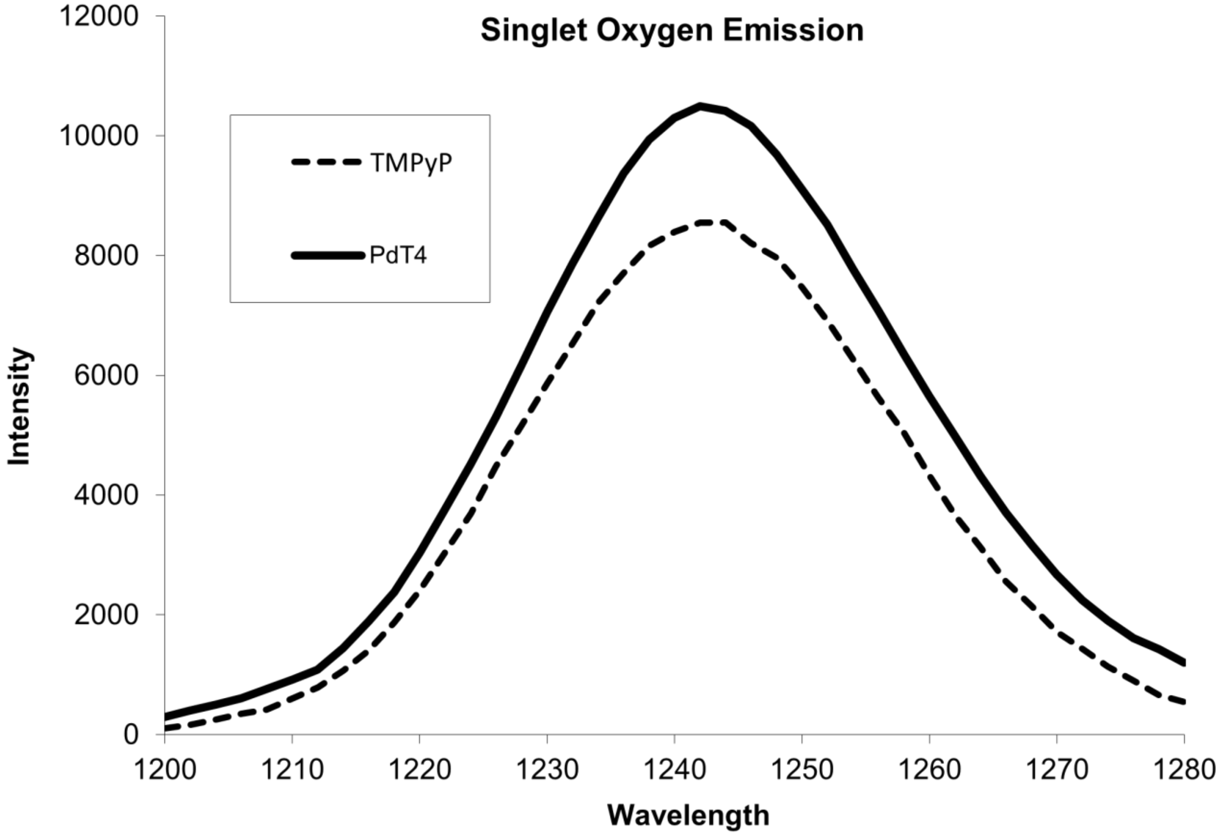

Figure S1. Singlet oxygen emission for TMPyP and PdT4.

Figure S2. Porphyrin and blue light control tests. Controls were conducted to evaluate bacteriophage inactivation from blue light or porphyrins independently. In the absence of porphyrin, blue light alone did not inactivate bacteriophages, as shown in Figure S2. Some minor inactivation of bacteriophage fr in TMPyP was observed in the porphyrin controls, as shown in Figure S3. Bacteriophage fr inactivation was likely due to genotoxicity of TMPyP, or the effect of ambient light, despite tests being conducted in a darkened room. For this reason, inactivation was quantified using the porphyrin controls as a baseline. However, no other bacteriophages were significantly inactivated by exposure to porphyrins in the absence of light.

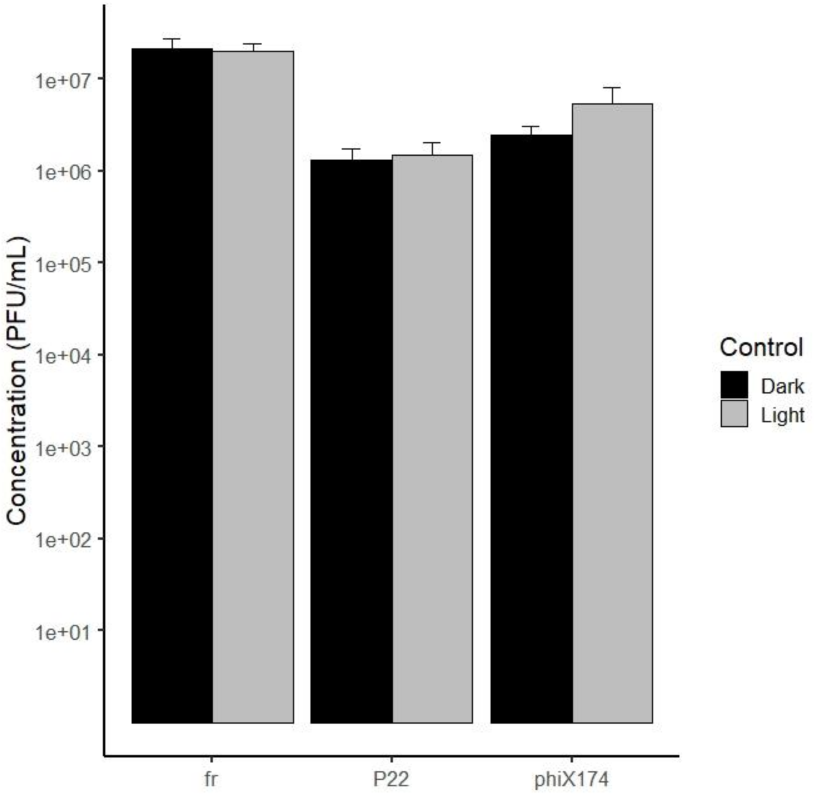

Figure S2. Bacteriophage concentrations after exposure to blue light (405 nm, 60 mW cm^{-2}). No bacteriophages were inactivated by blue light alone. Values represent means of triplicate independent experiments; error bars represent one standard deviation.

Figure S3. Bacteriophage concentrations after dark incubation with and without porphyrin (10 μM). Values represent means of triplicate independent experiments; error bars represent one standard deviation.

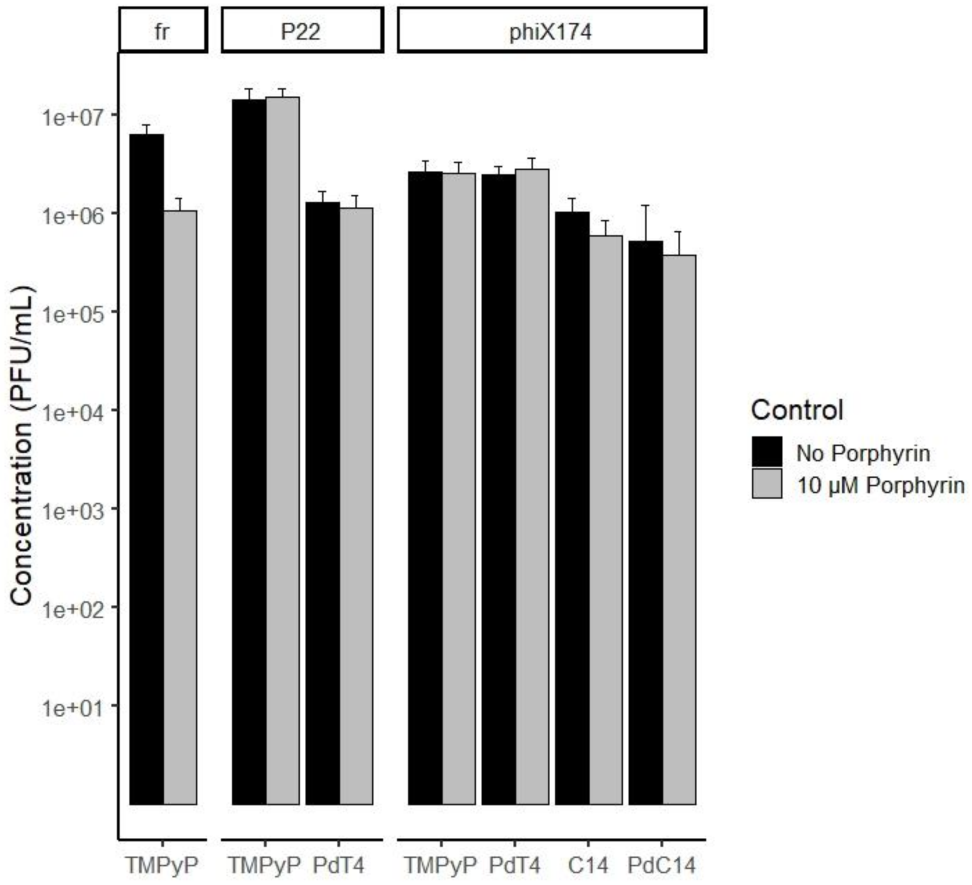

S3. Porphyrin/phage binding indicated by absorbance spectra of PdT4 and bacteriophages. Absorbance spectra of 10 μM PdT4 were measured with and without contact with bacteriophages, as shown in Figure S4. Though phage concentrations (~10^8 PFU/mL) were higher than those used for photoinactivation tests in this study, no apparent shift in the PdT4 absorbance was observed, even after 60 min dark incubation. However, at lower concentrations of PdT4 (2 μM), obvious shifts in the Soret peak were observed (see Figure 1 of the main text).

Figure S4. Absorbance spectra of PdT4 both alone ('None') and after dark incubation with bacteriophage ΦX174, P22, or fr at a HIGH PdT4:bacteriophage ratio (10 μM PdT4 : ~ 10^8 PFU/mL). Soret peaks (~417 nm) did not shift after contact with bacteriophages, indicating an excess of PdT4 at these concentrations.

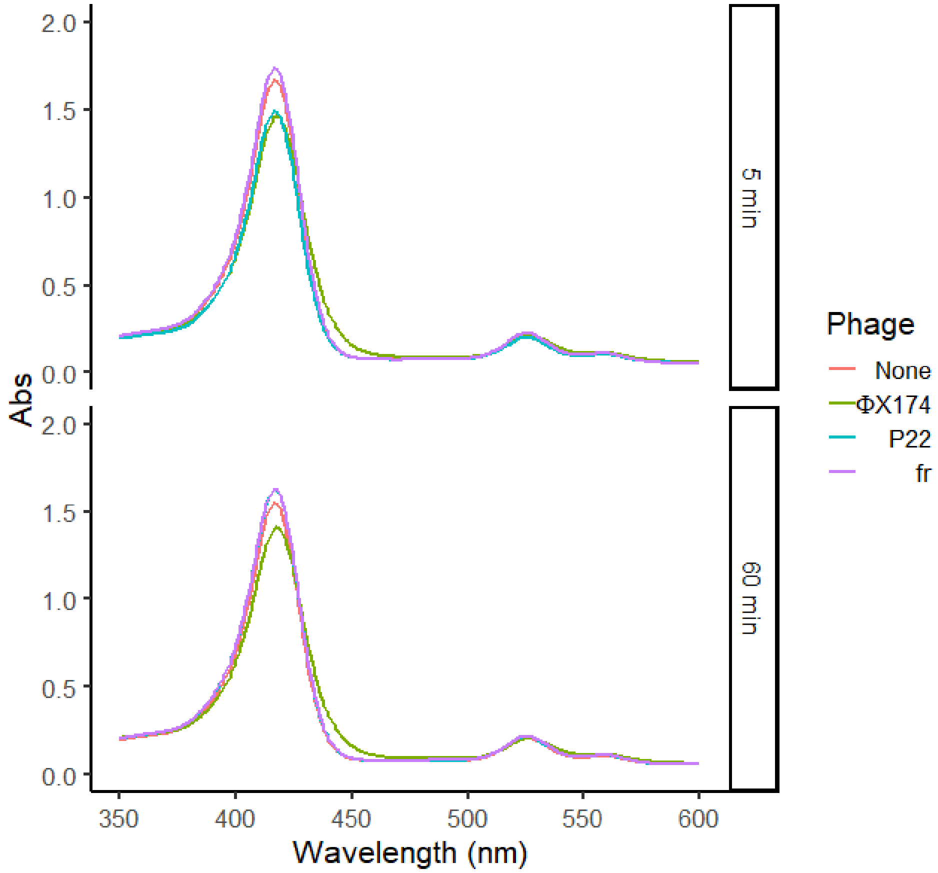

Absorbance spectra were subsequently used to assess the incubation time for porphyrin binding to viruses using a low PdT4:bacteriophage ratio of 2 μM PdT4 incubated with bacteriophages phiX174, P22, or fr (~ 10^{10} PFU/mL). A shift in the Soret peak was apparent after 5 min incubation, and no further shift was observed after 15 min incubation, as shown in Figure S5. Therefore, 5 min incubation was deemed sufficient to achieve porphyrin/virus contact. The results of the absorbance spectra confirmed observation of an immediate color change in porphyrin solutions on addition of high bacteriophage titers.

Figure S5. Absorbance spectra of PdT4 both alone (‘None’) and after incubation with bacteriophage ΦX174, P22, or fr at a LOW PdT4:bacteriophage ratio (2 μM PdT4 : ~ 10^{10} PFU/mL). Samples were incubated in the dark for 5 or 15 minutes prior to absorbance measurements. No additional shift in PdT4/phage Soret peaks was observed between 5 and 15 minutes.

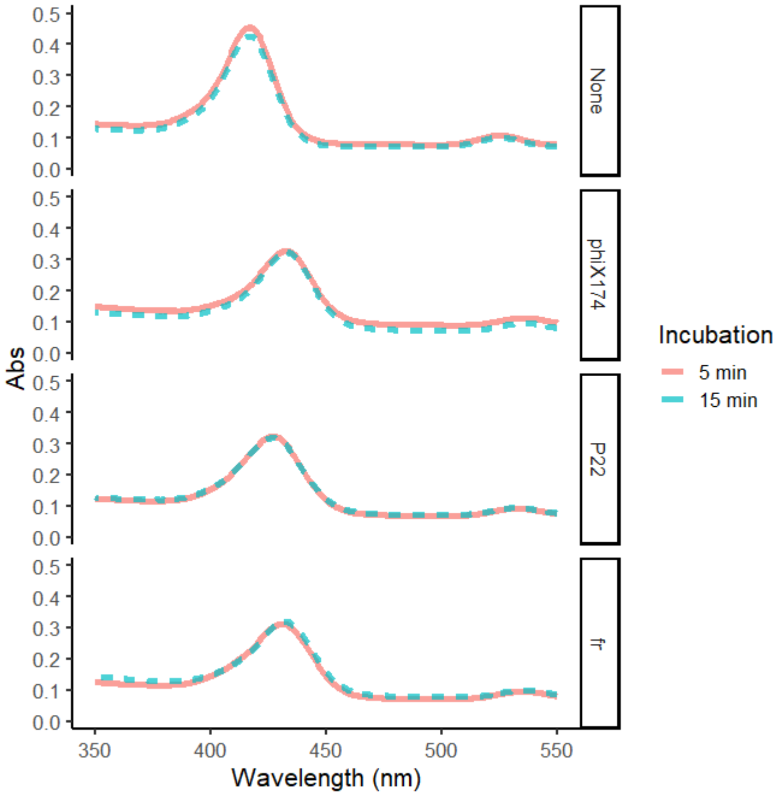

S4. Calculation of inactivation rate constants from the literature

To compare efficiency of photodynamic inactivation from reports in the literature, inactivation rates were estimated, as reported in Table 2 of the main text. Pseudo-1st order log_{10} inactivation rates were calculated assuming linear log reduction over reported exposure durations. If inactivation data were available at multiple exposures, the longest duration showing a linear semi-log trend in reduction over time was selected. Normalized rate constants were calculated by dividing pseudo-1st order rate constants by the concentration of porphyrin (μM) and the light intensity (mW cm^{-2}), thus giving an indication of relative treatment efficiency. All inactivation values were rounded to the nearest log_{10} removal to avoid reporting a misleadingly high level of precision.

Table S1. Photodynamic inactivation exposure and intensity values used to calculate approximate pseudo-1st order rate constants.

**Source**	**Virus**	**Compound**	**Intensity (mW cm^{-2})**	**Conc. (μM)**	**Exposure (min)**	**Approx. log_{10} removal (to nearest log)**	**Pseudo- 1st order rate constant (log_{10} s^{-1})**	**Normalized rate constant (log_{10} L cm^2 μmol^{-1} mJ^{-1})**
				Enveloped mammalian viruses				
Obrien 1992 [2]	Herpes simplex 1 virus	Merocyanine 540	7	26	4	4	2E-02	9E-05
Käsermann 1997 [3]	Vesicular stomatitis virus	Fullerene	29	1400 (1 mg/mL)	180	5	5E-04	1E-08
	Semliki Forest virus				180	5	5E-04	1E-08
Moor 1997 [4]	Vesicular stomatitis virus	AlPcS4	46	1	5	4	1E-02	3E-04
		Pc4		0.005	10	4	7E-03	3E-02
Silva 2005 [5]	Herpes simplex 1 virus	Pyridylvinyl-substituted tetraphenol porphryin	50	0.5	15	2	2E-03	9E-05
Peddinti 2008 [6]	Vesicular stomatitis virus	ZnTMPyP	80	1% wt film	60	6	2E-03	NA
Nikolaeva-Glomb	Influenza	Hemato-porphy	100	20	30	1	6E-04	3E-07

2017 [7]	virus A	rin						
	Bovine viral diarrhea virus					3	2E-03	8E-07
		GaPc1				2	1E-03	6E-07
		GaPc2				4	2E-03	1E-06
		InPc1				3	2E-03	8E-07
Remichkova 2017 [8]	Bovine viral diarrhea virus	ZnPcMe	100	0.58	5	2	7E-03	1E-04
	Herpes simplex 1 virus				5	3	1E-02	2E-04
	Vaccinia virus				5	2	7E-03	1E-04
	Newcastle disease virus				5	0	NA	NA
	Bovine viral diarrhea virus	ZnPcS			5	5	2E-02	3E-04
	Herpes simplex 1 virus				5	3	1E-02	2E-04
	Vaccinia virus				5	2	7E-03	1E-04
	Newcastle disease virus				5	1	3E-03	6E-05
Nonenveloped mammalian viruses								
Schagen 1999 [9]	Recombi-nant ade-novirus (E1 deficient)	Methylene blue	106	1.3	5	5	2E-02	1E-04
		Rose bengal		10	10	4	7E-03	6E-06
		Uroporphyrin		20	10	3	5E-03	2E-06
		AlPcS4		10	20	4	3E-03	3E-06
Peddinti 2008 [6]	Human adenovirus 5	ZnTMPyP4+	80	1% wt film	60	4	1E-03	NA
Nikolaeva-Glomb 2017 [7]	Human adenovirus 5	GaPc1	100	20	30	3	2E-03	8E-07

	Poliovirus 1				30	2	1E-03	6E-07
	Human adenovirus 5	GaPc2			30	4	2E-03	1E-06
	Poliovirus 1				30	1	6E-04	3E-07
	Human adenovirus 5	Hemato-porphyrin			30	2	1E-03	6E-07
	Poliovirus 1				30	1	6E-04	3E-07
	Human adenovirus 5	InPc1			30	2	1E-03	6E-07
	Poliovirus 1				30	1	6E-04	3E-07
Remichkova 2017 [8]	Cox-sackievirus B1	ZnPcMe	100	0.58	20	0	0	0
	Human adenovirus 5				20	1	8E-04	1E-05
Majiya 2018 [10]	Murine norovirus-1	TMPyP	32	5	20	2	2E-03	1E-05
				10	20	2	2E-03	5E-06
	Bovine enterovi-rus-2			5	60	1	3E-04	2E-06
				10	60	3	8E-04	3E-06
Nonenveloped bacteriophages								
Cho 2010 [11]	MS2 phage	Amine-functionalized fullerol	0.165	20	30	2	1E-03	3E-04
			0.19	10	30	2	1E-03	6E-04
Costa 2012 [12]	T4-like phage	Tri-Py+-Me-PF	40	5	90	4	7E-04	4E-06
	Aeromonas phage			5	90	1	2E-04	9E-07
	Vibrio phage			5	90	2	4E-04	2E-06
	Pseudo-monas phage			5	90	2	4E-04	2E-06
	MS2 phage			0.5	15	4	4E-03	2E-04
	Qbeta phage			0.5	15	4	4E-03	2E-04
	LAIST_PG0			0.5	15	6	7E-03	3E-04

	02							
Majiya 2018 [10]	Qbeta phage	TMPyP	32	0.5	6	6	2E-02	1E-03
Majiya 2019 [13]	MS2 phage	TMPyP	32	0.5	0.7	7	2E-01	1E-02
	phiX174 phage	TMPyP			6	1	4E-03*	7E-06
	P22 phage				1.5	3	4E-02*	6E-05
	fr phage				0.1	4	5E-01*	8E-04
This study	phiX174 phage	PdT4	60	10	1.5	1	1E-02*	2E-05
	P22 phage				0.4	3	1E-01*	2E-04
	phiX174 phage	C14			1.5	1	8E-03*	1E-05
		PdC14			3	2	1E-02*	2E-05

* Pseudo 1st-order rate constants were calculated from linear trend in multiple exposure values

References

1. Bork, M.A.; Gianopoulos, C.G.; Zhang, H.; Fanwick, P.E.; Choi, J.H.; McMillin, D.R. Accessibility and external versus intercalative binding to DNA as assessed by oxygen-induced quenching of the palladium(II)-containing cationic porphyrins Pd(T4) and Pd(t D4). *Biochemistry* **2014**, *53*, 714–724, doi:10.1021/bi401610t.
2. Obrien, B.J.M.; Gaffney, D.K.; Wang, T.P.; Sieber, F. Merocyanine 540-Sensitized Photoinactivation of Enveloped Viruses in Blood Products: Site and Mechanism of Phototoxicity. *Blood* **1992**, *80*, 277–285.
3. Käsermann, F.; Kempf, C. Photodynamic inactivation of enveloped viruses by buckminsterfullerene. *Antiviral Res.* **1997**, *34*, 65–70.
4. Moor, A.C.E.; Wagenaars-van Gompell, A.E.; Brand, A.; Dubbelmanl, T.M.A.R.; VanSteveninck, J. Primary Targets for Photoinactivation of Vesicular Stomatitis Virus by AlPcS4 or Pc4 and Red Light. *Photochem. Photobiol.* **1997**, *65*, 465–470.
5. Silva, E.M.P.; Giuntini, F.; Faustino, M.A.F.; Tome, P.C.; Silva, A.M.S.; Neves, M.G.P.M.S.; Tome, A.C.; Ferrer-correia, J.; Santana-marques, M.G.; Caeiro, M.F.; et al. Synthesis of cationic b-vinyl substituted meso-tetraphenylporphyrins and their in vitro activity against herpes simplex virus type 1. *Bioorg. Med. Chem.* **2005**, *15*, 3333–3337, doi:10.1016/j.bmcl.2005.05.044.
6. Peddinti, B.S.T.; Scholle, F.; Ghiladi, R.A.; Spontak, R.J. Photodynamic Polymers as Comprehensive Anti-Infective Materials: Staying Ahead of a Growing Global Threat. *ACS App.. Mater. Interfaces* **2018**, *10*, 25955–25959, doi:10.1021/acsami.8b09139.
7. Nikolaeva-Glomb, L.; Mukova, L.; Nikolova, N.; Kussovski, V.; Doumanova, L.; Mantareva, V.; Angelov, I.; Wohrle, D.; Galabov, A.S. Photodynamic Effect of some Phthalocyanines on Enveloped and Naked Viruses. *Acta Virol.* **2017**, *61*, 341–346, doi:10.4149/av.
8. Remichkova, M.; Mukova, L.; Nikolaeva-glomb, L.; Nikolova, N.; Doumanova, L.; Mantareva, V.; Angelov, I.; Kussovski, V.; Galabov, A.S. Virus inactivation under the photodynamic effect of phthalocyanine zinc(II) complexes. *Z. Naturforsch* **2017**, *72*, 123–128, doi:10.1515/znc-2016-0119.
9. Schagen, F.H.E.; Moor, A.C.E.; Cheong, S.C.; Cramer, S.J.; Ormondt, H. Van; Eb, A.J. Van Der Photodynamic treatment of adenoviral vectors with visible light : an easy and convenient method for viral inactivation. *Gene Ther.* **1999**, *6*, 873–881.
10. Majiya, H.; Adeyemi, O.O.; Herod, M.; Stonehouse, N.J.; Millner, P. Photodynamic inactivation of non-enveloped RNA viruses. *J. Photochem. Photobiol. B Biol.* **2018**, *189*, 87–94, doi:10.1016/j.jphotobiol.2018.10.009.
11. Cho, M.; Lee, J.; Mackeyev, Y.; Wilson, L.J.; Alvarez, P.J.J.; Hughes, J.B.; Kim, J.-H. Visible Light Sensitized Inactivation of MS-2 Bacteriophage by a Cationic Amine-Functionalized C60 Derivative. *Environ. Sci. Technol.* **2010**, *44*, 6685–6691, doi:10.1021/es1014967.
12. Costa, L.; Tomé, J.P.C.; Neves, M.G.P.M.S.; Tomé, A.C.; Cavaleiro, J.A.S.; Cunha, Â.; Faustino, M.A.F.; Almeida, A. Susceptibility of non-enveloped DNA- and RNA-type viruses to photodynamic inactivation. *Photochem. Photobiol. Sci.* **2012**, *11*, 1520–1523, doi:10.1039/c2pp25156f.
13. Majiya, H.; Chowdhury, K.F.; Stonehouse, N.J.; Millner, P. TMPyP functionalised chitosan membrane for efficient sunlight driven water disinfection. *J. Water Process Eng.* **2019**, *30*, 100475, doi:10.1016/j.jwpe.2017.08.013.
